# *HisB* as novel selection marker for gene targeting approaches in *Aspergillus niger*

**DOI:** 10.1186/s12866-017-0960-3

**Published:** 2017-03-08

**Authors:** Markus R. M. Fiedler, Tarek Gensheimer, Christin Kubisch, Vera Meyer

**Affiliations:** Institute of Biotechnology, Department of Applied and Molecular Microbiology, Technische Universität Berlin, Gustav-Meyer-Allee 25, 13355 Berlin, Germany

**Keywords:** *Aspergillus niger*, Gene expression, Selection marker, HisB, Tet-on

## Abstract

**Background:**

For *Aspergillus niger*, a broad set of auxotrophic and dominant resistance markers is available. However, only few offer targeted modification of a gene of interest into or at a genomic locus of choice, which hampers functional genomics studies. We thus aimed to extend the available set by generating a histidine auxotrophic strain with a characterized *hisB* locus for targeted gene integration and deletion in *A. niger*.

**Results:**

A histidine-auxotrophic strain was established via disruption of the *A. niger hisB* gene by using the counterselectable *pyrG* marker. After curing, a *hisB* ^*-*^ *, pyrG* ^*-*^ strain was obtained, which served as recipient strain for further studies. We show here that both *hisB* orthologs from *A. nidulans* and *A. niger* can be used to reestablish histidine prototrophy in this recipient strain. Whereas the *hisB* gene from *A. nidulans* was suitable for efficient gene targeting at different loci in *A. niger*, the *hisB* gene from *A. niger* allowed efficient integration of a Tet-on driven luciferase reporter construct at the endogenous non-functional *hisB* locus. Subsequent analysis of the luciferase activity revealed that the *hisB* locus is tight under non-inducing conditions and allows even higher luciferase expression levels compared to the *pyrG* integration locus.

**Conclusion:**

Taken together, we provide here an alternative selection marker for *A. niger*, *hisB*, which allows efficient homologous integration rates as well as high expression levels which compare favorably to the well-established *pyrG* selection marker.

**Electronic supplementary material:**

The online version of this article (doi:10.1186/s12866-017-0960-3) contains supplementary material, which is available to authorized users.

## Background

The filamentous fungus *Aspergillus niger* is an industrially exploited cell factory with a broad product portfolio including primary metabolites, proteins and enzymes [1]. Recent data proved that *A. niger* can also serve as a suitable host for secondary metabolite production [2, 3]. Additionaly, *A. niger* is a model system used to study fundamental molecular and cellular processes. Various selection systems are available for transformation of *A. niger*, including nutritional (*pyrG*, *trpC*, *amdS, niaD*, *sC, agaA* and *argB*) and antibiotic resistance (*hph, ble*) markers [4–11]. Recently this set was expanded by two new nutritional markers (*nicB* and *adeA*) which can be used for gene deletion in *A. niger* [12]. However, in order to study the function and interplay of several genes, or to construct/re-engineer a complete metabolic pathway in *A. niger*, it is of advantage having a range of selection markers at hand to choose the best one suited for a given approach. The number of nutritional markers for *A. niger* is limited to seven, as recently published [12] and although marker recycling using the Cre/*loxP* system has been established for Aspergilli [13, 14], they often suffer from poor recombination events ranging from 5 to 20%. So far, within the set of nutritional selection markers available for *A. niger*, only the *pyrG* and *agaA* loci [4, 15, 16] meet the need of a well characterized locus for efficient homologous integration of single copy expression cassettes. In fungi, a transcriptionally active, non-protein encoding locus that is targeted by exogenous expression cassettes at high frequency has been an important molecular technique underlying transformation, mutant complementation, and functional genomic approaches to study gene function [17]. Even the highly efficient CRISPR/Cas system which has been applied recently for filamentous fungi [17–21], depends on well characterized loci for genomic integration of the genes of interest.

In order to establish an alternative auxotrophic selection marker for *A. niger*, which can also be used for both gene targeting or insertion of an expression cassette into a well characterised locus, we chose the histidine biosynthesis pathway as a target. This pathway was intensively studied in *Salmonella typhimurium*, *Escherichia coli* and *Corynebacterium glutamicum* (for reviews see [22, 23]) and in *A. nidulans* [24]. It generates histidine in ten reaction steps catalysed by seven enzymes in a branched pathway out of phosphoribosyl pyrophosphate supplied via the pentose phosphate pathway. As it was shown that deletion of *hisB* in *A. nidulans* and A*. fumigatus* results in histidine auxotrophy [25, 26], we selected *hisB*, which catalyses the sixth step in the histidine biosynthesis pathway and disrupted it via a direct targeting approach in *A. niger*. Subsequent integration of the well-established Tet-on system [27] using luciferase as reporter gene enabled us to evaluate gene expression characteristics at the *hisB* locus in comparison to the widely used *pyrG* locus. We could furthermore demonstrate that genome editing using the *hisB* orthologue of *A. nidulans* is feasable.

## Methods

### Strains, growth conditions and molecular techniques

*A. niger* strains used in this study are listed in Table 1. The strains were grown at 30°C in minimal medium (MM) [28] or complete medium (CM), consisting of MM supplemented with 1% yeast extract and 0.5% casamino acids. 10 mM uridine or 10 mM histidine were added to the medium when required.

**Table 1:** *A. niger* strains used in this work

Name	Genotype	Reference
N402	*cspA*	[38]
MA169.4	*kusA::DR-amdS-DR, pyrG* ^*−*^ (AB4.1 derivative)	[15]
MF40.6	*kusA::DR-amdS-DR, pyrG* ^*+*^ *, hisB::ThisB-AopyrG-ThisB* (MA169.4 derivative)	this study
MF41.3	*kusA::DR-amdS-DR, pyrG* ^*-*^ *, hisB* ^*-*^ (MF40.6 derivative)	this study
MF42.2	*kusA::DR-amdS-DR, hisB* ^*-*^ *pyrG* ^*+*^ (MF41.3 derivative)	this study
MF43.1	*kusA::DR-amdS-DR, pyrG* ^*+*^ *, olvA::AnidhisB,* (MF42.2 derivative)	this study
MF44.1	*kusA::DR-amdS-DR, pyrG* ^*+*^ *, olvA* ^*+*^ *, AnidhisB* ^*+*^ (transformed with pSE1.6, MA42.2 derivative)	this study
AW8.4	*kusA::DR-amdS-DR, olvA::AopyrG*	[33]
MA169.4 *pyrG* ^*+*^	*kusA::DR-amdS-DR, AopyrG* ^*+*^ *, olvA* ^*+*^(transformed with pAW34, MA169.4 derivative)	this study
TG1.14	*kusA::DR-amdS-DR*, *Tet*﻿*-*﻿*on (single copy)* (MF42.2 derivative)	this study
TG2.3	*kusA::DR-amdS-DR*, *Tet* *-* *on* *-mluc (single copy)* (MF42.2 derivative)	this study
VG7.2	*pyrG* ^*+*^, *Tet* *-* *on * * (single copy)* (AB4.1 derivative)	[27]
VG8.27	*pyrG* ^*+*^, *Tet* *-* *on* *-mluc (single copy)* (AB4.1 derivative)	[27]

To obtain *pyrG* ^*-*^ strains via counterselection, 2 x 10^7^ spores were plated on MM plates containing 75 mg/ml 5-Fluoroorotic acid (FOA), 10 mM uridine, 10 mM proline and 10 mM histidine. Plates were incubated at 30°C for 1-2 weeks until single colonies were visible. FOA-resistant mutants were purified on MM + FOA plates once and tested for their uridine auxothrophy on MM plates containing 10 mM histidine or 10 mM histidine and 10 mM uridine, respectively.

All molecular techniques were performed according to standard procedures [29] and the transformation, genomic DNA extraction and Southern blot were performed as described elsewhere [30].

### *Construction of a* hisB *disruption vector*

To construct a *hisB* disruption plasmid we used an approach which was published recently [31]. In brief, 533 bp and 500 bp sequences of the *hisB* coding and 3’ sequence were amplified via PCR using primers listed in Additional file 1: Table S1. Both fragments were inserted via Gibson cloning into the BsrGI linearized plasmid pAO4-13 carrying the *A. oryzae pyrG* gene [32] giving rise to the counter-selectable *hisB* disruption plasmid pMF22.1.

### *Construction of an* olvA *deletion cassette*

The plasmid pAW34 ([33], kindly provided by Arthur Ram) containing the *AopyrG* gene flanked by the 5’ and 3’ region of *olvA* was used as a backbone. The *A. nidulans hisB* (AN6536) gene was amplified using primers listed in Additional file 1: Table S1 and cloned into the XhoI/HindIII linearized pAW34 via Gibson cloning giving rise to plasmid pSE1.6.

### Construction of luciferase reporter constructs

The *A. niger* pyrG* gene within Tet-on plasmids pVG2.2 (containing the empty Tet-on system, [27]) and pVG4.1 (containing a codon optimized version of the luciferase *mluc* under control of the Tet-on system, [27]) was replaced by a 2291 bp fragment amplified by fusion PCR, containing the full length *hisB* gene without a functional start codon flanked by 5’ and 3’ region of the *hisB* gene, giving rise to pTG1.2 and pTG2.15, respectively (Additional file 2: Figure S2).

### Measurement of the luciferase activity

Ninety six well microtiter plate assays were performed as described earlier [27] with slight modifications. In brief, 5 x 10^4^ spores were inoculated in 200 μl CM medium [30] supplemented 1.4 mM luciferin and 0, 5 (A) or 20 μg/ml (B) doxycycline in a microtiter plate and incubated at 30°C in a Victor^3^ (Perkin Elmer). OD and luminescence were measured every 30 min.

## Results and discussion

**Fig. 1:**
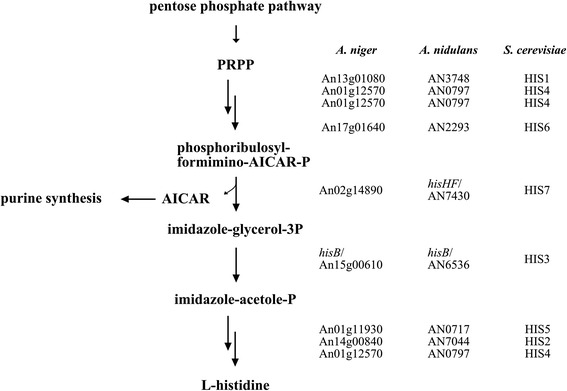
Pathway of histidine biosynthesis in *A. niger* according to the KEGG database [39] including respective ORF codes or protein names of proteins for *A. niger*, *A. nidulans* [40] and *S. cerevisiae* [41]

**Fig. 2:**
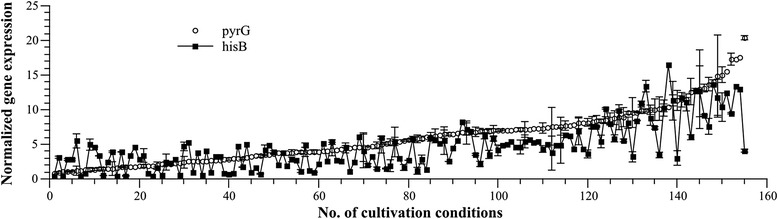
Expression profile of the *A. niger pyrG* and *hisB* genes. Our in-house transcriptomic database for *A. niger* covering 155 growth conditions was analyzed for *hisB* and *pyrG* expression levels. Gene expression levels were normalized against actin (*actA*) expression during the exponential growth phase of *A. niger* in maltose-based bioreactor cultivation. For improved visualization, data were assorted ascending for the *pyrG* expression level

In order to construct an alternative auxotrophic marker for *A. niger*, we choose the *A. niger* orthologue of the well characterised *S. cerevisiae* selection marker HIS3. The gene codes for an imidazole-glycerol-phosphate dehydratase which catalyses the sixth step of the histidine biosynthesis in *S. cerevisiae*, by specifically dehydrating imidazole-glycerol-3-phosphate, producing imidazole-acetole-phosphate (Fig. 1). Proteins Blasts of the HIS3 sequence against translated *A. niger* ORFs [34] revealed a single orthologue (An15g00610) for HIS3 in the genome of *A. niger* with a protein sequence identity of 57.6%, which was termed *hisB*. It is well known that gene expression depends on both position effects and on the availability of transcription factors [35]. We thus analysed whether gene expression levels of *hisB* and the widely used *pyrG* are comparable by scrutinzing an in-house database, which comprises genome-wide expression profiles of *A. niger* from 155 different cultivation conditions [36]. As depicted in Fig. 2, *hisB* is expressed under all conditions covered in the transcriptomic database in a range comparable to the *pyrG* gene (Fig. 2).

**Fig. 3:**
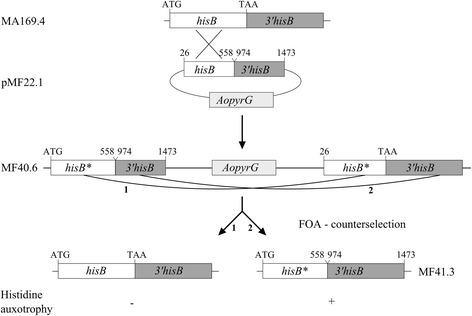
Schematic overview of the *hisB* disruption approach in *A. niger* and subsequent counterselection. The plasmid pMF22.1 was constructed via amplifying parts of the *hisB* gene and its 3’ region (numbers above the fragments indicate the DNA position from the start codon). Both fragments were inserted via Gibson cloning into the BsrGI linearized plasmid pAO4-13 carrying the *Aspergillus oryzae pyrG* [32] giving rise to the plasmid pMF22.1. The plasmid was transformed into the strain MA169.4 [15] thereby disrupting the *hisB* locus giving rise to MF40.6. Counterselection may either lead to a genomic rearrangement, thereby eliminating the plasmid via recombination in its *hisB* locus or the 5’ region, creating a truncated hisB* (MF41.3) or wildtype *hisB* loci, respectively

We used a gene disruption approach published earlier [31] to inactivate the *hisB* gene. A part of the open reading frame of the *hisB* gene was integrated together with the 3’ region of *hisB* into pOA4-13 which contains the *Aspergillus oryzae pyrG* gene as selection marker [32] giving rise to plasmid pMF22.1. Subsequent transformation of the vector into a *pyrG* ^*-*^ strain lacking a functional non-homologues-end-joining-pathway (NHEJ) [30], resulted in a single recombination event whereby the *hisB* gene became disrupted (Fig. 3). Counterselection on plates containing fluoroorotic acid (FOA) (see Methods) allowed recombination events between the duplicated parts of the *hisB* gene, thereby restoring gene functionality (Fig. 3). Alternatively, recombination between the 3’ regions resulted in a nonfunctional truncated *hisB* (hisB*) (Fig. 3). In total, 62 primary transformants were obtained from which 10 were randomly selected and tested for histidine auxotrophy. One of four transformants unable to grow on minimal medium (MM) lacking histidine (MF40.6) was counterselected as described in Material and Methods, resulting in 30 *pyrG* cured strains, which were purified and tested for their histidine auxotrophy. Only 10 strains out of 30 were found to be auxotrophic for histidine (Additional file 3: Figure S3, A), although the duplicated parts of the *hisB* gene and the 3’ regions did only differ in 33 bp in length. From the set of strains which were screened for histidine and uridine auxotrophy, strain MF41.3 (*hisB* ^*-*^ *, pyrG* ^*-*^) was chosen for further analysis. The auxotrophy was stable under non-selective conditions, which was proven by several rounds of cultivation on complete medium supplemented with 10 mM histidine and uridine. After three rounds, MF41.3 still remained unable to grow on MM lacking uridine or histidine (data not shown), which is in good agreement with earlier work published for *A. nidulans* and *A. fumigatus*, which showed that deletion of *hisB* leads to auxotrophic strains, which can be rescued by the addition of 1-5 mM histidine to the medium, thereby restoring wildtype-like growth [25, 26]. The truncated hisB* locus in MF41.3 was subsequently sequenced and compared to the *hisB* locus of MA169.4, showing the expected DNA modifications (Fig. 3 and Additional file 4: Figure S4). In order to exclude polygenetic effects in subsequent phenotypic analysis caused by deletion of two essential auxotrophic gene markers, we restored the *pyrG* gene in MF41.3 by transforming this strain with linearized plasmid pAB4.1 [7], containing the full length *A. niger pyrG* gene, giving strain MF42.2. This strain was able to grow on plates lacking uridine and the correct integration of the linearized fragment was confirmed via Southern blot analysis (data not shown).

To evaluate whether the *A. nidulans hisB* gene (AN6536) could be used as selection marker to complement the histidine auxotrophy, the genomic and protein sequences were compared using BlastN and BlastP, revealing a high conservation (90.2%) on the protein level, whereas the nucleotide sequence conservation was considerably lower (74.1%), which was thought to be crucial for the correct integration of the deletion cassette into the region of choice instead of complementing *A. niger hisB* gene at its endogenous locus. For an easy read-out of the transformation and gene replacement efficiency, the *olvA* gene was chosen. This gene encodes a hydrolase involved in DHN-melanin synthesis in *A. niger* and its deletion results in an incomplete melanin biosynthesis and thus green spore formation [33].

In doing so, plasmids pSE1.6 (*olvA::AnidhisB*) and pAW34 (*olvA::AopyrG*) were linearized and transformed into MF42.2 (*hisB* ^*-*^, *pyrG* ^*+*^) or MA169.4 (*pyrG* ^*-*^), respectively. Analysis of total transformants and spore color analysis revealed that deletion of *olvA* with the *A. nidulans hisB* gene leads to a deletion efficiency comparable to the *pyrG* marker (Table 2). Strains carrying the *A. nidulans hisB* gene at either the *olvA* locus or ectopically integrated into the genome were selected, purified, spotted on CM and compared to the *olvA::AopyrG* strain AW8.4 and MA169.4 with an ectopically integrated copy of pAW34. No macroscopic differences were detected between mutant or wild type strains except spore color formation (Additional file 5: Figure S5).

**Table 2:** Homologous recombination efficiency of individual transformations, as assessed by phenotypic spore color screening using *A. oryzae pyrG* and *A. nidulans hisB* as selection markers to delete the *olvA* gene in *kusA* ^*-*^ recipient strains MF42.2 and MA169.4

Recipient strain	Transformed plasmid	No. of transformants	No. of positive mutants	Homologous integration efficiency [%]
MF42.2 (*hisB* ^*-*^ *, pyrG* ^*+*^)	pSE1.6 (*AnidhisB*)	34	30	91.2
MA169.4 (*pyrG* ^*-*^)	pAW34 (*AopyrG*)	31	27	89 ± 1.9
		90	81	
		35	30	
		21	19	

**Fig. 4:**
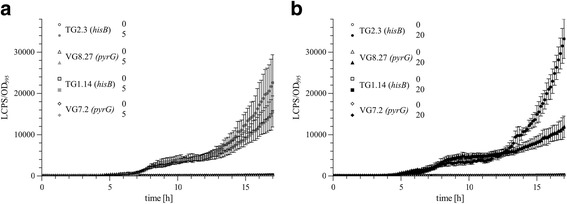
Luciferase activity assay of strains containing the *Tet-on::mluc* construct integrated into the hisB* or *pyrG* loci. The luciferase activity assay was performed as described earlier [27] ﻿using 5 μg/ml (panel **a**) or 20 μg/ml (panel **b**) doxycycline for induction. Strains TG1.14 and VG7.1 are negative-control strains (not expressing luciferase but harboring the empty Tet-on constructs), while strains TG2.3 and VG8.27 carry the *Tet-on::mluc* constructs integrated at the *hisB* or *pyrG* locus, respectively. Mean values of triplicate experiments are shown

A luciferase based reporter system was used to test whether the truncated hisB* locus is suitable to allow gene expression of a gene of interest. In doing so, the luciferase reporter constructs pTG1.2 (containing the empty Tet-on system) and pTG2.15 (containing a codon optimized version of the luciferase *mluc* under control of the Tet-on system [27, 37]) were constructed. Both plasmids should integrate into the truncated hisB* locus via a single recombination event, because the *A. niger hisB* gene was used as selection marker, thereby restoring its functionality (Additional file 2: Figure S2). Transformation of MF42.2 resulted in 53 primary transformants for pTG1.2 and 22 primary transformants for pTG2.15, respectively. For both strains, 10 out of 20 transformants were proven by diagnostic PCR to harbor the expression constructs at hisB*, which was further confirmed via Southern analysis (Additional file 2: Figure S2). Transformants TG1.14 (carrying a single copy of *Tet-on::hisB*), TG2.3 (carrying a single copy of *Tet-on::mluc::hisB*) were selected and their luciferase activity determined and compared to the previously published strains VG7.2 and VG8.27 [21], harboring the Tet-on::pyrG* or Tet-on-mluc::pyrG* constructs at the *pyrG* locus, respectively (Fig. 4).

While the luminescence activity of the strains carrying the reporter construct integrated into the *pyrG* or *hisB* locus are comparable (induction with 5 μg/ml doxycycline), the values of the TG2.3 are 3 times higher when using 20 μg/ml doxycycline, possibly reflecting a higher transcriptional activity at the *hisB* locus under the conditions used. It is notable that neither the vector control (TG1.14, 20 μg/ml doxycycline) nor the non-induced luciferase constructs (TG2.3 and VG8.27) showed any luciferase activity during the experiment, clearly demonstrating that the system is tight at the *hisB* locus in the absence of the inducer.

## Conclusion

In summary, we report here a straight forward approach to rationally generate auxotrophic markers in the filamentous fungus *A. niger* which was employed to create a histidine auxotrophic strain which can be used as a recipient isolate for endogenous deletion of genes using the *A. nidulans* orthologue *hisB*. In addition, we characterized the hisB* locus for functionality to integrate expression constructs, which revealed an expression level for the luciferase reporter with a higher performance and tighter characteristics under non-induced conditions compared to the well-used *pyrG* locus. The tools described here significantly increase the tractability of *A. niger* at the molecular level and suggest *hisB* could be used for similar applications in other model or pathogenic filamentous fungi.

## Additional files

Additional file 1: Table S1. Primers used in this study. (DOC 36 kb)
Additional file 2: Figure S2. Schematic overview of the integration of the luciferase constructs into the *hisB** locus and confirmation via Southern analysis. (TIFF 42235 kb)
Additional file 3: Figure S3. Analysis of histidine auxotrophy of strains MF41.1-30. (TIFF 19712 kb)
Additional file 4: Figure S4. Sequencing results of the *hisB** locus. (TIFF 16195 kb)
Additional file 5: Figure S5. Growth comparison of *olvA* ^*-*^ and *olvA* ^*+*^ mutants with *AnidhisB* or Ao*pyrG* background on CM plates. (TIFF 7346 kb)
