# Supplementary material for: HisB as novel selection marker for gene targeting approaches in Aspergillus niger
Source: BMC Microbiol. 2017 Mar 8;17:57. doi: 10.1186/s12866-017-0960-3 (PMC5343542; doi:10.1186/s12866-017-0960-3)
Supplement: Additional file 1: Table S1. — Primers used in this study. (DOC 36 kb) [file 12866_2017_960_MOESM1_ESM.doc]

**Additional file 1: TABLE S1:** Primers used in this study.

| **ORF code** | **Gene** | **Primer** | **Sequence (5’ to 3’ oriented)** | **Target** |
| --- | --- | --- | --- | --- |
| An15g00610 | *hisB* | his3_fragmentfw | tcttagagtggggtatcactaatgtacaAATCACGCGCATAACCAACG | 533 bp *hisB* fragment |
|  |  | his3_fragmetrev | ggacccgggGGCCGACTCGACAGATCAAT | 533 bp fragment |
|  |  | thisfragmentfw | gagtcggccCCCGGGTCCAGTATGCTTTT | 500 bp promoter *hisB* fragment |
|  |  | thisfragmentrev | tttgtacgatagtgaccgactgtacaGGCAGTCCCGCATATAACCA | 500 bp terminator *hisB* fragment |
|  |  | fullhispJet_fw | ctcgagtttttcagcaagatTGTATTCATCGACTCTGCGC | Complete *hisB* including promoter and terminator of *hisB* |
|  |  | fullhispJet_rev | ttgtaggagatcttctagaaagatACAGGCAGTCCCGCATATAACCA | Complete *hisB* including promoter and terminator of *hisB* |
|  |  | his*teton1new | ttggctccacgcgggcgcgccTGTATTCATCGACTCTGCGC | 967 bp promoter *hisB* |
|  |  | his*teton2new | gggaggggTTTGGTGGGTGTGCTTTTTTT | 967 bp promoter *hisB* |
|  |  | his*teton3new | acacccaccaaaCCCCTCCCCGCGCGAAC | 1324 bp *hisB** terminator *hisB* |
|  |  | his*_tetOn_rev | cggtggcggccgcaaggcgcgccGCAGTACGTCTCACTCCTCT | 1324 bp *hisB* terminator *hisB* |
| AN6536 | *hisB* | An_his3_olva_fw | accgataataaccatcctttaagcttCAAGGCTGCGTGAGGTTATC | *A. nidulans hisB* including promoter and terminator region |
|  |  | Anhis3_olva_rev | agcaagcaaacacactcgagCTCTTTCCGCAGCCTTCAAG | *A. nidulans hisB* including promoter and terminator region |
