# Supplementary figures and images for: HisB as novel selection marker for gene targeting approaches in Aspergillus niger
Source: BMC Microbiol. 2017 Mar 8;17:57. doi: 10.1186/s12866-017-0960-3 (PMC5343542; doi:10.1186/s12866-017-0960-3)

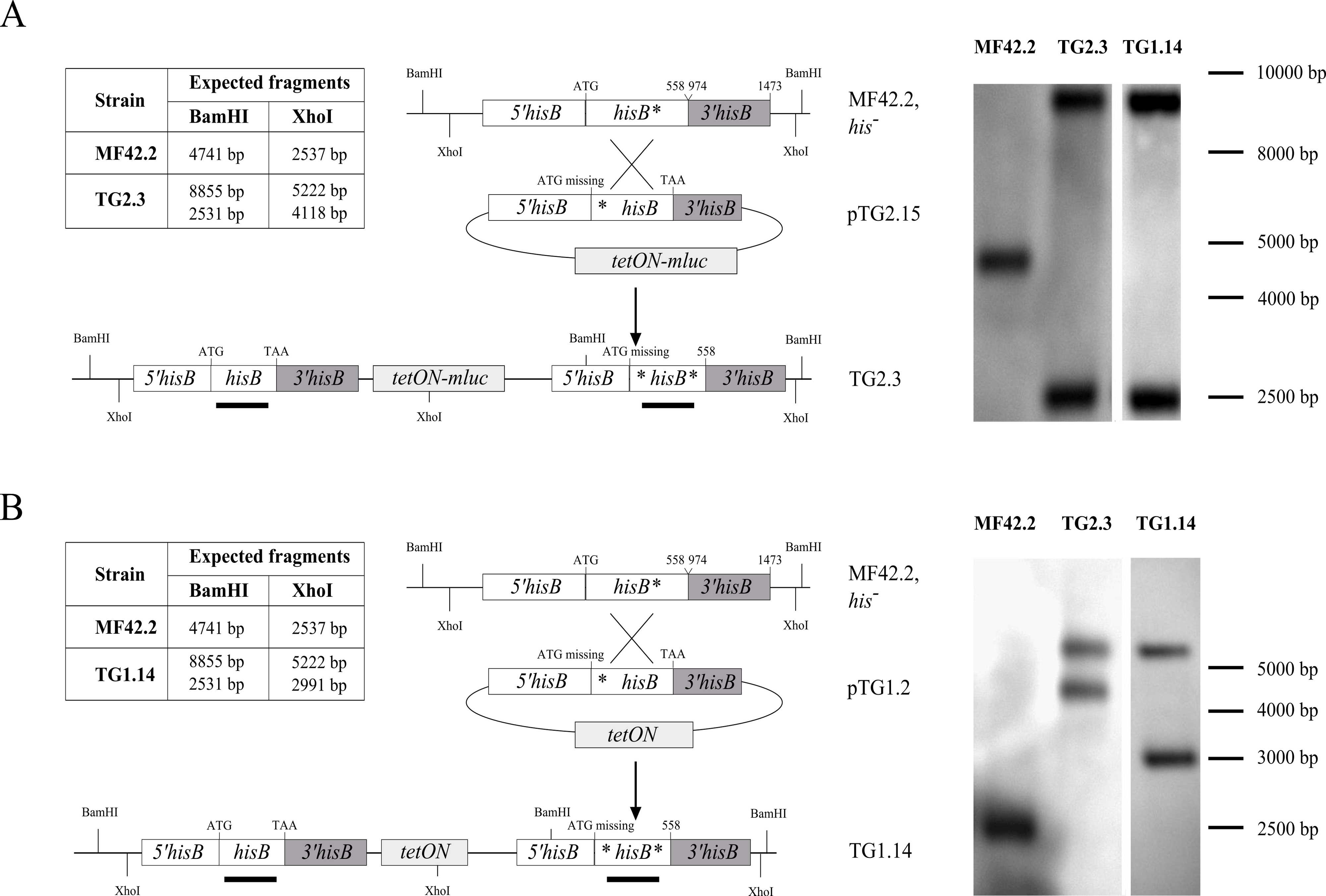

Supplement: Additional file 2: Figure S2. — Schematic overview of the integration of the luciferase constructs into the hisB* locus and confirmation via Southern analysis. (TIFF 42235 kb) [file 12866_2017_960_MOESM2_ESM.tiff]

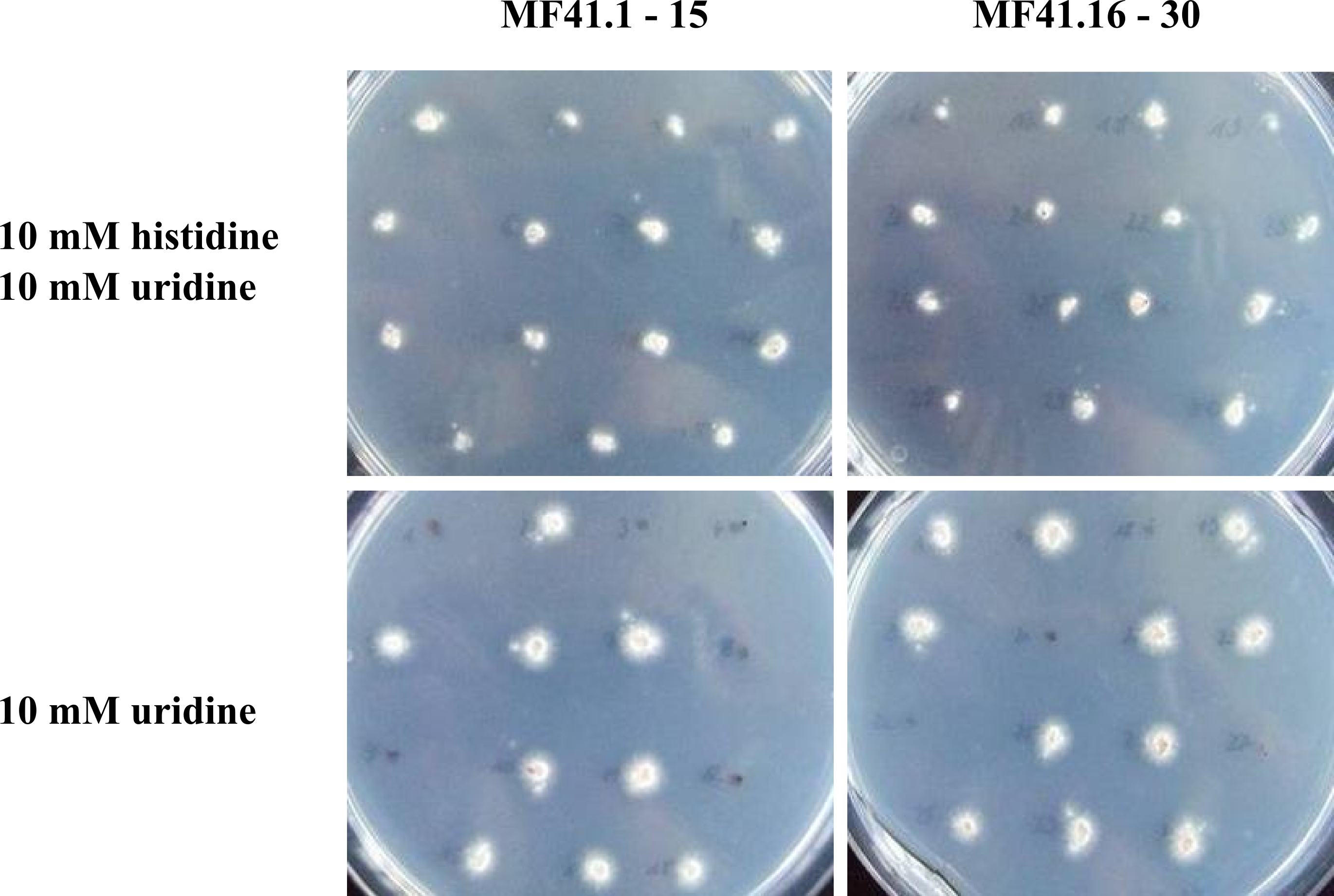

Supplement: Additional file 3: Figure S3. — Analysis of histidine auxotrophy of strains MF41.1-30. (TIFF 19712 kb) [file 12866_2017_960_MOESM3_ESM.tiff]

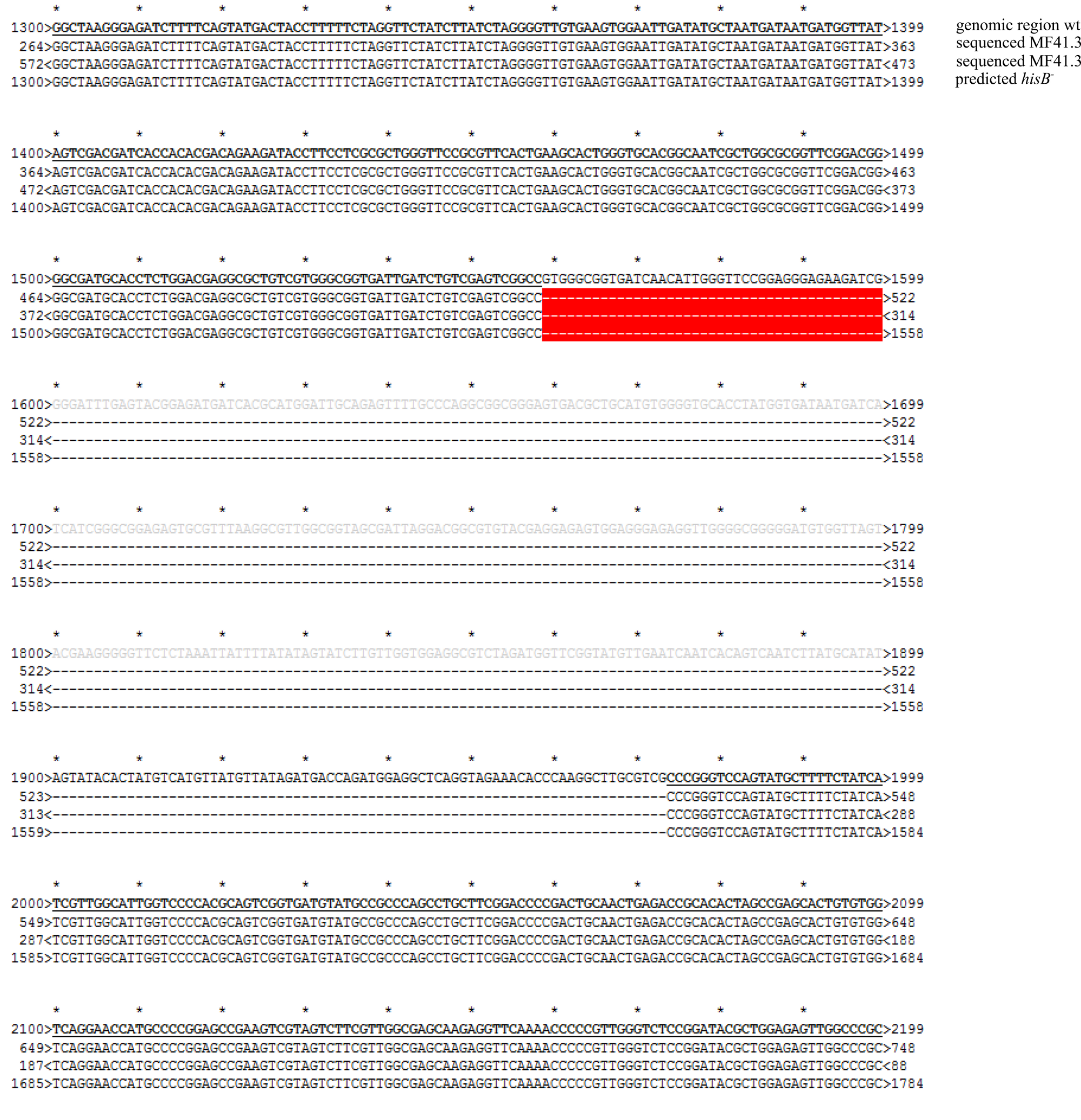

Supplement: Additional file 4: Figure S4. — Sequencing results of the hisB* locus. (TIFF 16195 kb) [file 12866_2017_960_MOESM4_ESM.tiff]

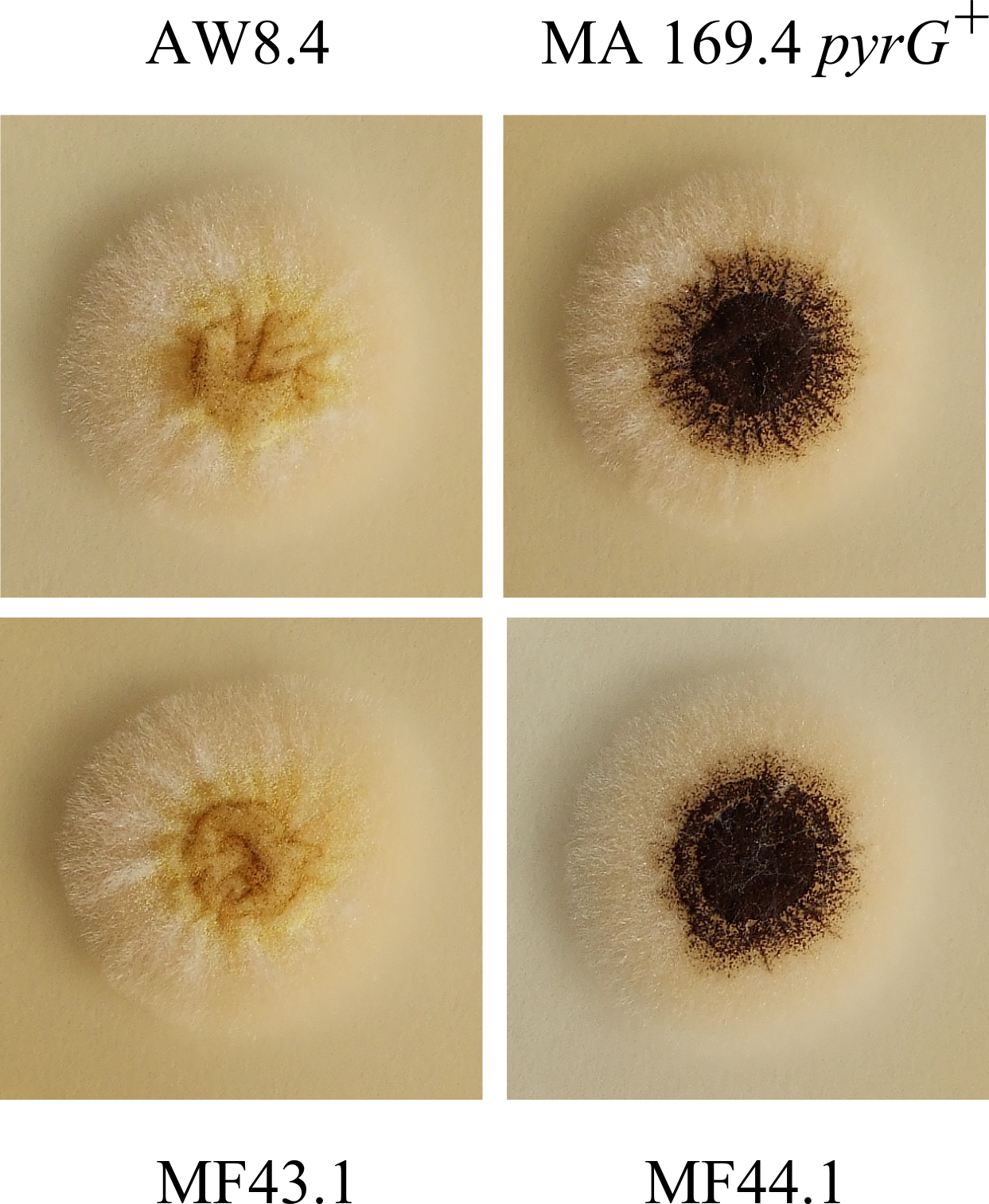

Supplement: Additional file 5: Figure S5. — Growth comparison of olvA - and olvA + mutants with AnidhisB or AopyrG background on CM plates. (TIFF 7346 kb) [file 12866_2017_960_MOESM5_ESM.tiff]
